# TMT-based quantitative proteomics reveals the genetic mechanisms of secondary hair follicle development in fine-wool sheep

**DOI:** 10.1371/journal.pone.0315637

**Published:** 2025-02-06

**Authors:** Li-Xia Qiu, Qian Yu, Hua-Qian Zhou, Wen-Hua Fan, Jing-Jing Zheng, Yong-Lin Yang, Wen-Zhe Zhang, Xin Cao, Hua Yang

**Affiliations:** 1 College of Animal Science and Technology, Northwest Minzu University, Lanzhou, Gansu, China; 2 State Key Laboratory of Sheep Genetic Improvement and Healthy Production, Xinjiang Academy of Agricultural and Reclamation Science, Shihezi, Xinjiang, China; 3 College of Animal Science and Technology, Shihezi University, Shihezi, Xinjiang, China; Affiliated Hospital of Jiangsu University, CHINA

## Abstract

The development of secondary hair follicles influences the quality of sheep wool. However, the mechanism by which proteins mediate the fetal development of secondary hair follicles remains unknown. In this study, the histomorphology of secondary hair follicles was analyzed over four stages of fetal development (75, 85, 95, and 105 gestational days). TMT-based quantitative proteomics was used to compare the differential protein profiles of skin tissues between consecutive developmental periods (75 versus 85, 85 versus 95, and 95 versus 105 gestational days). We found that the density of secondary hair follicles and the secondary hair follicles/primary hair follicles ratio increased from 85 to 105 gestational days. Bioinformatic analysis identified 238, 35, and 348 differentially expressed proteins in the respective comparison periods. Focal adhesion, ECM−receptor interaction, and the estrogen signaling pathway all played important roles in secondary hair follicle development. COL1A1, THBS3, ITGA6, COL6A1, and THBS4 were identified as potential candidate proteins in the initiation of secondary hair follicles. This study provides valuable proteomics data on secondary hair follicle development and thus has deepened our understanding of the molecular mechanisms underlying wool quality traits in fine-wool sheep.

## Introduction

Fine-wool sheep play an important role in animal husbandry and economic development in the pastoral areas of the central and western regions of semi-agricultural China. Wool is a high-quality natural protein fiber with good hygroscopic properties that is both lightweight and soft, and it is used to make clothes with excellent heat retention qualities. Hair follicles (HFs) are skin appendages located within the dermis, and they are the only organ that develops periodically and undergoes cyclic regeneration throughout the lives of mammals. HF development affects aspects of wool yield and quality, including fiber diameter (FD) and length. According to the timing of development and morphological characteristics, HFs can be classified as primary hair follicles (PFs) and secondary hair follicles (SFs). SFs can further develop into re-differentiated SFs. It has been demonstrated previously that in Chinese Merino sheep (Junken type), the development of PFs is initiated at 65 d; SFs develop at 85 d, and re-differentiated SFs develop at 105 d of gestation [1]. The development of SFs determines the quantity and quality of the wool; it most notably determines the FD, an important index of wool quality and the most important factor determining the price of wool [2].

Previous studies have focused on the molecular regulatory mechanisms of HF development, and several differentially expressed genes, mRNAs, LncRNAs, miRNAs, and proteins have been identified. In particular, miR-195, *CHP1*, *SMAD2*, *FZD6*, and *SIAH1* were related to HF initiation and development [3]. Shang et al. used high-throughput sequencing to identify 153 miRNAs and 29 signaling pathways related to SF development in the fetal skin tissues of Inner Mongolia cashmere goats, including the chi-miR-26b-5p, chi-miR-495-3p, Wnt, and TGF-β signaling pathways [4]. Sulayman et al. studied the expression profiles of lncRNA and mRNA in sheep at four time points in embryonic development (E65, E85, E105, and E135), and identified 471 lncRNAs and 12,812 mRNAs in 37-day-old lambs using RNA-seq technology [5]. Previously, 10 candidate miRNAs were identified as important regulators of SF initiation in fetal skin samples from Chinese Merino sheep [6]. Proteins belonging to the keratin family are highly expressed in the anagen and catagen follicular phases; the estrogen and ECM-receptor interaction signaling pathways are specifically involved in SF development [7]. Guo et al. identified 227 specific co-expressed proteins and 123 differentially expressed proteins (DEPs) in the fetal skin tissues of Gansu alpine fine-wool sheep at four developmental stages (E87, E96, E102, and E138) using label-free proteomics [8]. Notably, the proportions of KRT85, KRTAP15-1, and KRTAP3-1 may be the key factors determining the FD [9]. While certain DEPs have been identified in the HF life cycle and development in fetal sheep, the mechanisms of protein regulation involved in SF development remain unknown.

Chinese Merino sheep (Junken type) represent one of the fine wool sheep breeds in China, where wool production remains an important agricultural commodity. Recent studies have investigated the genetic mechanisms underlying the wool traits of Chinese Merino sheep, and SNPs related to wool traits have been identified [10, 11]. In particular, *DKK1* regulates HF morphogenesis and cycling, [12] and *SHCBP1* influences wool crimping [13]. The expression profiles of miRNAs in SF initiation have been studied during different development periods [6]. However, the protein expression patterns and DEPs during SF development in the sheep fetus remain unclear. To understand the regulatory mechanisms guiding HF development and wool quality, we identified DEPs associated with SF development in Chinese Merino sheep (Junken type) using histomorphology, TMT-based quantitative proteomics, and bioinformatic analysis.

## Materials and methods

### Animals and sample preparation

This study was carried out in accordance with the Guidelines for the Care and Use of Laboratory Animals and approved by the Experimental Animal Care and Use Committee of Xinjiang Academy of Agricultural and Reclamation Sciences (Shihezi, China, ethics committee approval number: XJNKKXY-2020-34, December 30, 2020). All efforts were made to minimize animal suffering.

Fifty 2.5-year-old healthy female super fine-wool Chinese Merino sheep (Junken type) with a mean FD of 17.5 ± 0.5 μm were selected and mated with the rams of the same breed (FD of 16.6 ± 0.5 μm) by artificial insemination at the sheep breeding farm of the Xinjiang Academy of Agricultural and Reclamation Sciences. The day of insemination was designated as embryonic day 0 (E0). Three fetuses were collected at each of four sampling dates (75d (E75), 85d (E85), 95d (E95), and 105d (E105)) of gestation according to known periods of HF development [5, 6, 8, 14]. On the day of sampling, the pregnant ewes were exsanguinated after being stunned using a captive-bolt gun. The uterus was removed, and the fetuses were obtained. The fetuses were washed in phosphate-buffered saline and exsanguinated. Skin samples were collected from the two sides of each fetus and then divided into two parts; one part was frozen in liquid nitrogen for proteomics analysis, and the other was fixed in 4% paraformaldehyde for HF histomorphology.

### Preparation and staining of paraffin sections

The skin tissues in each stage were trimmed into 1 cm^2^ blocks, washed with 1× PBS buffer, and placed in 4% paraformaldehyde for fixation over 24 h. The fixed tissue blocks were washed with 1× PBS buffer and dehydrated by a graded ethanol series, cleared with xylene, embedded in paraffin, and sectioned using a microtome (Leica RM2235, Leica Microsystems, Heidelberg, Germany); tissue slices were 7 μm thick, with transverse slices and longitudinal slices taken according to the HF growth direction. The slices were fixed for 1 h and dried at 37°C. H&E staining was performed to detect the morphological changes in HFs.

### Calculation of HF density

The HF tissue sections were observed under a microscope (Nikon Eclipse TE2000-U Kanagawa, Japan), and images were obtained with Nikon NIS Elements software. The visual field area was calculated according to the scale of the slice, and the PF density, SF density, and the ratio of SF density to PF density (S/P) were calculated from E85 to E105. The data were presented as the mean ± standard error (SE). The differences in the number and density of HFs were analyzed between groups using SPSS 25.0 (IBM Corp., Armonk, NY, USA).

### Tissue protein extraction and concentration determination

Skin tissues were pulverized and frozen in liquid nitrogen, and total protein was extracted with 8 M urea, 0.1% SDS, and the protease inhibitor PMSF followed by ultrasonic emulsification. The protein concentration was measured using a BCA Protein Assay Kit (Thermo Scientific, Waltham, MA, USA), and SDS-PAGE was performed with 12% (w/v) acrylamide discontinuous gels. Electrophoresis of proteins was performed at 90 V for 40 min for the 12% sample gels, then at 120 V for 120 min for the separating gels. The dye used for visualizing proteins was Coomassie Brilliant Blue R250.

### TMT labeling and chromatographic column sample classification

Protein samples (100 μg) were taken from each skin section and digested with trypsin by FASP. The liquid was collected and dried after enzymatic hydrolysis. Peptide samples (100 μg) were obtained from each sample for labeling. The sample processing and labeling were completed according to the protocols of a Pierce TMT^®^ Mass Tagging Kit and a Reagent Kit; the mixed labeled samples were used for C18 column classification. The classification was performed according to standard HPLC procedures. The TMT-labeled samples were vacuum concentrated and dried at 45°C, dissolved in 500 μL of A solution (2% ACN, 98% H_2_O, PH10, NH_4_OH), and the supernatant was collected after centrifugation. The sample outflow liquid was collected, and then gradient elution was performed to collect the components every minute using a 1.5 mL centrifuge tube; adjacent 2 min samples were combined according to the chromatogram to form a total of 20 components.

### Liquid chromatography–tandem mass spectrometry (LC–MS/MS) analysis

After full dissolution, each sample was injected into the LC–MS/MS system for analysis using a Thermo Scientific Q Exactive mass spectrometer (Thermo Scientific, Waltham, MA, USA). Liquid chromatography phase A consisted of 99.9% water and 0.1% formic acid, and phase B consisted of 99.9% acetonitrile and 0.1% formic acid. The scan range was 350 to 1600 m/z. The parent ion of ionic strength TOP25 was selected for secondary mass spectrometry identification. The parent ions were fragmented by HCD, and the secondary mass spectrometry sequence was determined. The ratio of reported ions was quantified to generate the original mass spectrometry data files.

### Quantitative proteomic analysis

The MS/MS data were processed using Proteome Discoverer 1.4 (Thermo Scientific, Waltham, MA, USA) against the UniProt sequence database (*Ovis aries*) for peptide identification and quantification. The qualitative results of peptide and protein mass spectrometry were obtained at 1% FDR.

### Bioinformatic analysis

The proteomics data were analyzed using an omics data analysis tool. To determine the biological processes involved in SF development and the functional relevance of DEPs, Gene Ontology (GO) analysis was used to assign the DEPs to biological process (BP), cell component (CC), and molecular function (MF) categories using the DAVID database. The Kyoto Encyclopedia of Genes and Genomes (KEGG) was employed to identify enriched high-level functions in the defined biological systems using the DAVID database and the KOBAS database. Then, the results of the GO and KEGG pathway analyses were visualized using R software. A protein-protein interaction (PPI) network was constructed from the DEPs related to SFs and mapped using STRING Ver.11.0 (https://string-db.org/). The PPI network was visualized using Cytoscape v3.9.1.

### Real-time PCR verification

To verify the TMT-based results from E75 to E105 in SF development, the transcript-level expression levels of 10 candidate genes were confirmed using SYBR Green fluorescence-based qPCR; the primer sequences are listed in Table 1. Total RNA of skin samples from E75, E85, E95, and E105 was extracted using a Total RNA Kit I (Omega, Norcross, GA, USA), and the total RNA was retrotranscribed with One-Step gDNA Removal and cDNA Synthesis Super Mix (TRANS, Beijing, China) in the presence of oligo (dT)18 primers according to the manufacturer’s instructions. qPCR was carried out with a LightCycler® 480 II (Roche, Mannheim, Germany) using the LightCycler® 480 SYBR Green I Master (Roche, Mannheim, Germany). Each qPCR sample was run in a 20 μL total volume comprising 10 μL of 2×SYBR Green I Master qPCR Mix, 0.8 μL of 10 μM forward and reverse primers, 6.4 μL of water, and 2 μL of cDNA template. The PCR conditions were as follows: 5 min at 94°C followed by 45 cycles at 98°C for 10 s and 60°C for 10 s (different genes were run under slightly different cycle conditions), with a final extension at 72°C for 10 s. A melting curve was generated to confirm the identity of each PCR product. The experiments were performed in triplicate with double-distilled water as the negative control.

**Table 1:** Primer sequences and PCR product sizes.

Gene	Accession No.	Primer Sequence (5’-3’)	Product Size (bp)	Annealing temperature (°C)
*GAPDH*	NM_001190390.1	F: CTGACCTGCCGCCTGGAGAAA R: GTAGAAGAGTGAGTGTCGCTGTT	149	60
*VTN*	XM_027975239.2	F: GCAGAGACCACGGGAACCT R: CCCTTGCAGGACTCTTGGTC	128	60
*THBS3*	XM_004002577.4	F: AATCCCACCCAGACAGATGC R: CCAAGTCCGTCGTTGTCTG	152	60
*THBS4*	XM_027971492.2	F: CTGCAGCCAATCCTGACAG R: GGTAACGGAGGACGGCTT	172	60
*MAP1B*	XM_015101253.3	F: CTTCGCCGAGGCTTAACGG R: CCATGATCGGATTCCAAGCTC	226	59
*HPX*	XM_004016210.5	F: TGGGGCTTTGATGCTACCAC R: CCCAGAATTTGTCCCCCTTG	193	60
*TPMT*	XM_012101237.4	F: GACAGCGCACGAGACATT R: GTCAGCACCCGGTCTTTCTG	148	60
*COL1A1*	XM_027974705	F: AGTGTGCCCCAGAAGAACTG R: GCCGTACTCGAACTGGAATC	102	60
*KRT27*	NM_001114763.2	F: TGACGACCTTAGGAACCAGAT R: GCTCTGATGAAGGGCCTGTT	133	59
*KRT25*	NM_001009739.1	F: TGAACGTGGAGATGAACGCA R: CAGGGAGTGTTTCGTGGCTA	263	58
*KRT34*	XM_004012898.5	F: CAGCGAGGACAGCAAG R: TGGGCTTGTTCTAGGC	147	58

### Western blot analysis

The protein samples of sheep skins were prepared by using RIPA buffer (high) (R0010, Solarbio, Beijing, China). Then, the supernatant was collected and the protein concentration was measured by BCA method. The extracted proteins were mixed with a 5×loading buffer (Epizyme, Shanghai, China) and boiled at 100°C for 10 min to denature the proteins, then subjected to 10% SDS-PAGE, electrotransferred onto the polyvinylidene difluoride membrane (PVDF, Millipore IPVH00010, Solarbio), and blotted as described previously [15]. The antibodies are as follows: GAPDH (AF7021, diluted 1:3000; Affinity, Jiangsu, China), TPMT (110682-1-AP, diluted 1:800; Proteintech, Wuhan, China), COL1A1 (667288-1-lg, diluted 1:5000; Proteintech, Wuhan, China). Primary antibody binding was visualized using secondary antibodies and soaked in the enhanced chemiluminescence detection reagent (ECL kit, Thermo Fisher Scientific, Waltham, Massachusetts USA), and scaned using ChemiDoc XRS + image analyzer (Bio-Rad, California, USA). GAPDH was used as the internal control, and the test was repeated three times.

### Data analysis

One-way ANOVA was conducted to evaluate the changes over the four fetal stages. The effects of single treatments were analyzed with Tukey’s test for multiple comparisons at the same probability level after ANOVA using SPSS25.0. For the qPCR data analysis, the relative expression levels of candidate genes were calculated using the 2^-ΔΔCt^ method.

## Results

### Histomorphological analysis of SF development

**Fig 1:**
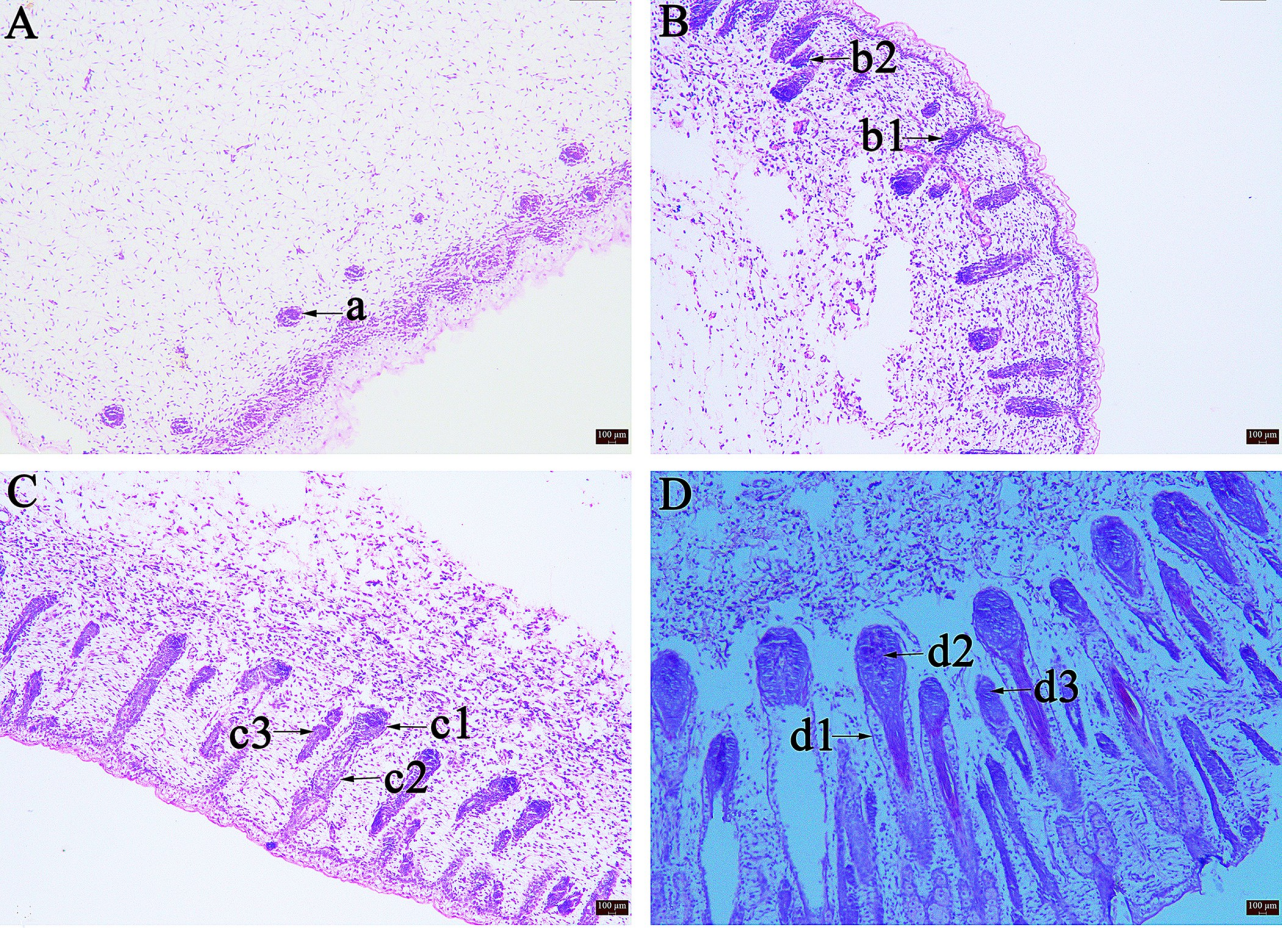
The morphological changes during HF development from E75 to E105. (A) E75(100×); (B) E85(100×); (C) E95(100×); (D) E105(100×) a: Hair germ cells, b1: PFs, b2: SFs, c1: Hair bulb, c2: PFs, c3: SFs, d1: Outer root sheath, d2: Dermal papilla, d3: Re-differentiated SFs.

Morphological differences in HF development could be observed from E75 to E105 (Fig 1). At E75, the proliferation of PFs began from the stratum germinativum to the dermis, and the hair buds began to develop (Fig 1A). At E85, the PFs grew from the dermis to the epidermis and formed hair fiber tubes that filled the germinal matrix. At the same time, the SFs began to develop, and hair bulbs (primordia) of the SFs were generated around the PFs (Fig 1B). At E95, the HFs continued to develop; the numbers of hair fiber tubes from PFs and SFs were increased, and the hair dermal papillae were formed (Fig 1C). At E105, the PFs continued to grow and passed through the epidermis, while the SFs developed further and formed the secondary-derived re-differentiated SFs that gradually grew upwards and began to form the hair. The hair bulb cells continued to differentiate. At the same time, the sebaceous glands were gradually forming (Fig 1D).

### Density of HFs

Analysis of the density and S/P values of HFs from E85 to E105 revealed that as the SFs appeared and propagated, the oldest SFs underwent differentiation to become re-differentiated SFs (Table 2). The PF density was significantly higher on E95 compared to E85 and E105 (*p* < 0.01). The density of PFs exhibited an initial increase followed by a subsequent decrease as fetal development progressed, reaching a peak by E95. In contrast, the SF density and S/P values were significantly higher on E105 compared to E95 and E85 (*p* < 0.01), both of which continuously increased with fetal development and reached a maximum on E105; there were no differences in S/P values between E85 and E95 (*p* > 0.05).

**Table 2:** HF density and S/P ratio from E85 to E105.

Fetal period	PF (number/mm^2^)	SF (number/mm^2^)	S/P
E85	133.11±0.73^B^	77.26±0.63^C^	0.58±0.02^B^
E95	142.27±0.43^A^	115.29±0.43^B^	0.81±0.05^B^
E105	79.48±0.35^C^	157.47±0.29^A^	1.96±0.08^A^

Note: The values with different uppercase letters in the same row indicated significant differences at *p* < 0.01.

### SDS-PAGE

**Fig 2:**
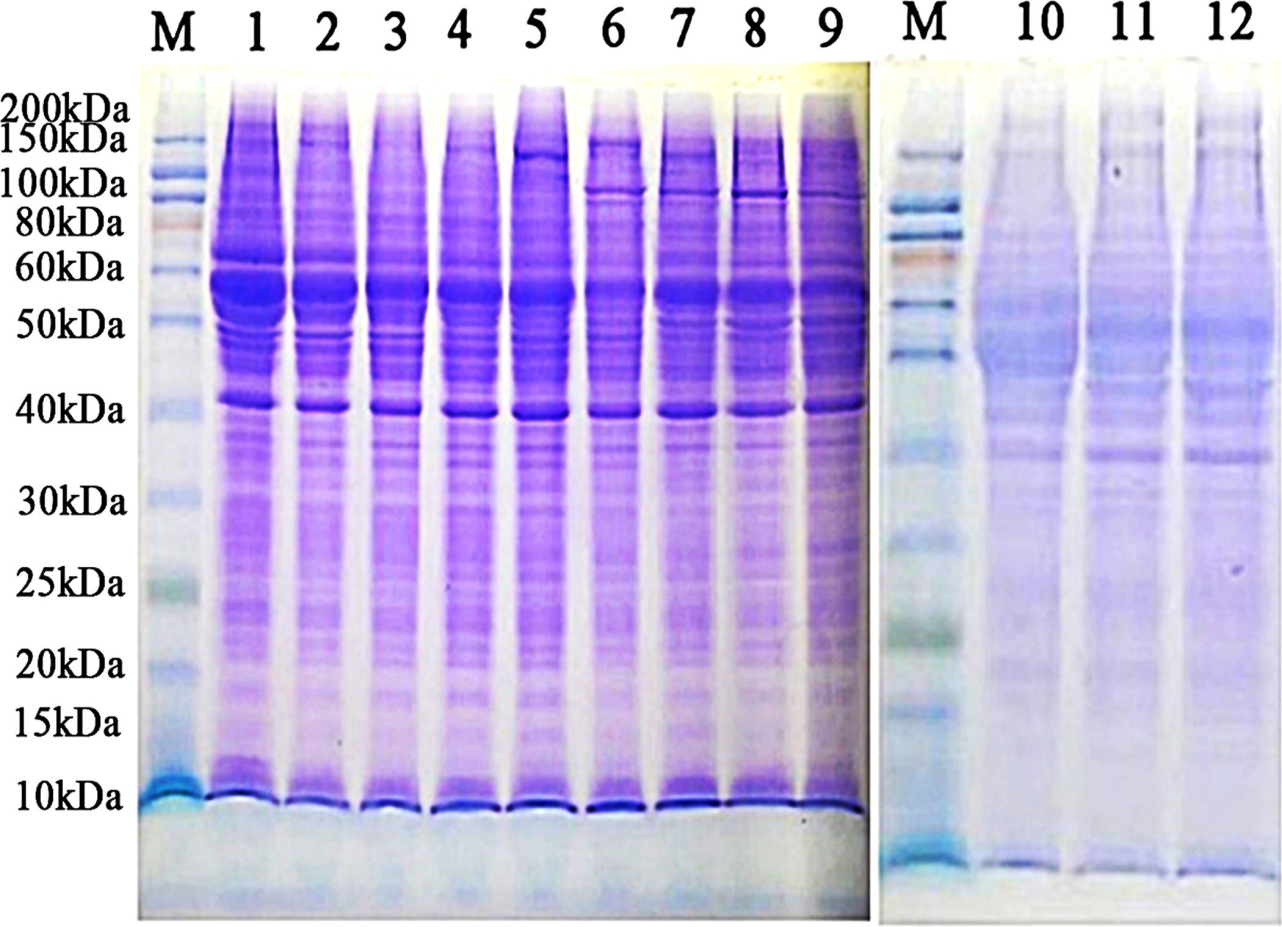
SDS-PAGE gel electrophoresis. M: the protein molecular weight standard. 1–3: E75. 4–6: E85. 7–9: E95. 10–12: E105.

SDS-PAGE revealed that the molecular weights of each tissue protein band were between 40 and 50 kDa; the protein concentration exceeded 2 mg/mL, and the extracted protein was of good quality and suitable for proteomic analysis (Fig 2).

### Overview of proteomics sequencing

Total protein obtained from skin tissues derived from the four fetal periods E75, E85, E95, and E105 were separately run and analyzed in triplicate for LC-MS/MS identification. Quality control was performed to check the MS data, and the results indicated that the data satisfied the prerequisites for subsequent advanced analysis (S1 Table).

Proteins containing at least one unique peptide were defined as trusted proteins, accounting for 74% of the total identified proteins. The proteins with a coverage of 0–50% accounted for 92% of the trusted proteins. The relative molecular masses of proteins were largely distributed between 10 and 60 KDa, and those above 120 kDa accounted for 71% of the trusted proteins. These specifications indicated that the proteome data obtained in this experiment were reliable and of high quality.

### Identification of DEPs at four stages of development

**Fig 3:**
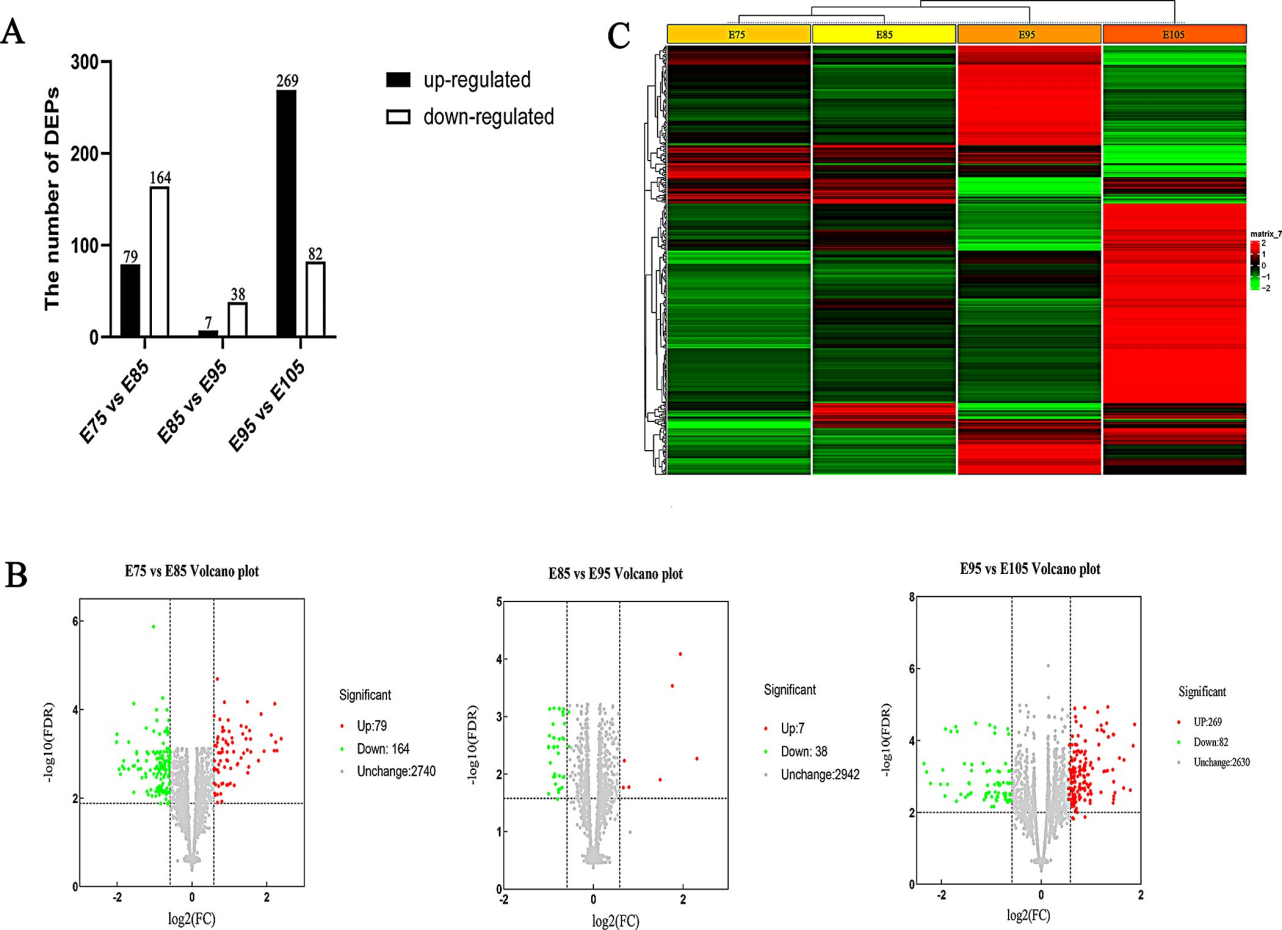
DEPs analysis. (A) The number of DEPs in three comparison pairs. (B) Volcano plot of DEPs. (C) A heatmap displaying the DEPs at four different stages (E75, E85, E95, and E105). Red: relatively high expression; Green: relatively low expression.

After filtering to remove missing values and normalization, 4,994 proteins remained, and 4,974, 4,991, 4,989, and 4,994 proteins were identified as belonging to E75, E85, E95, and E105, respectively (S1 Table). A total of 509 DEPs were recruited using the criteria of fold change (FC) > 1.5 and T-test Q-value ≤ 0.05 [8, 16]. This included 79 up-regulated and 164 down-regulated proteins in the comparison of E75 vs E85. Additionally, seven proteins were up-regulated and 38 proteins were down-regulated in the E85 vs E95 comparison; 269 proteins were up-regulated and 82 proteins were down-regulated in the E95 vs E105 comparison (Fig 3A). Volcano plots of −Log 10 (FDR) versus log 2 (FC) were then generated (Fig 3B). A hierarchical cluster analysis for DEPs was conducted using R 4.2.1 (Fig 3C). The DEPs in the four developmental periods were clustered into two categories and three subcategories; E75 and E85 were clustered into one category.

### GO and KEGG pathway enrichment of DEPs

**Fig 4:**
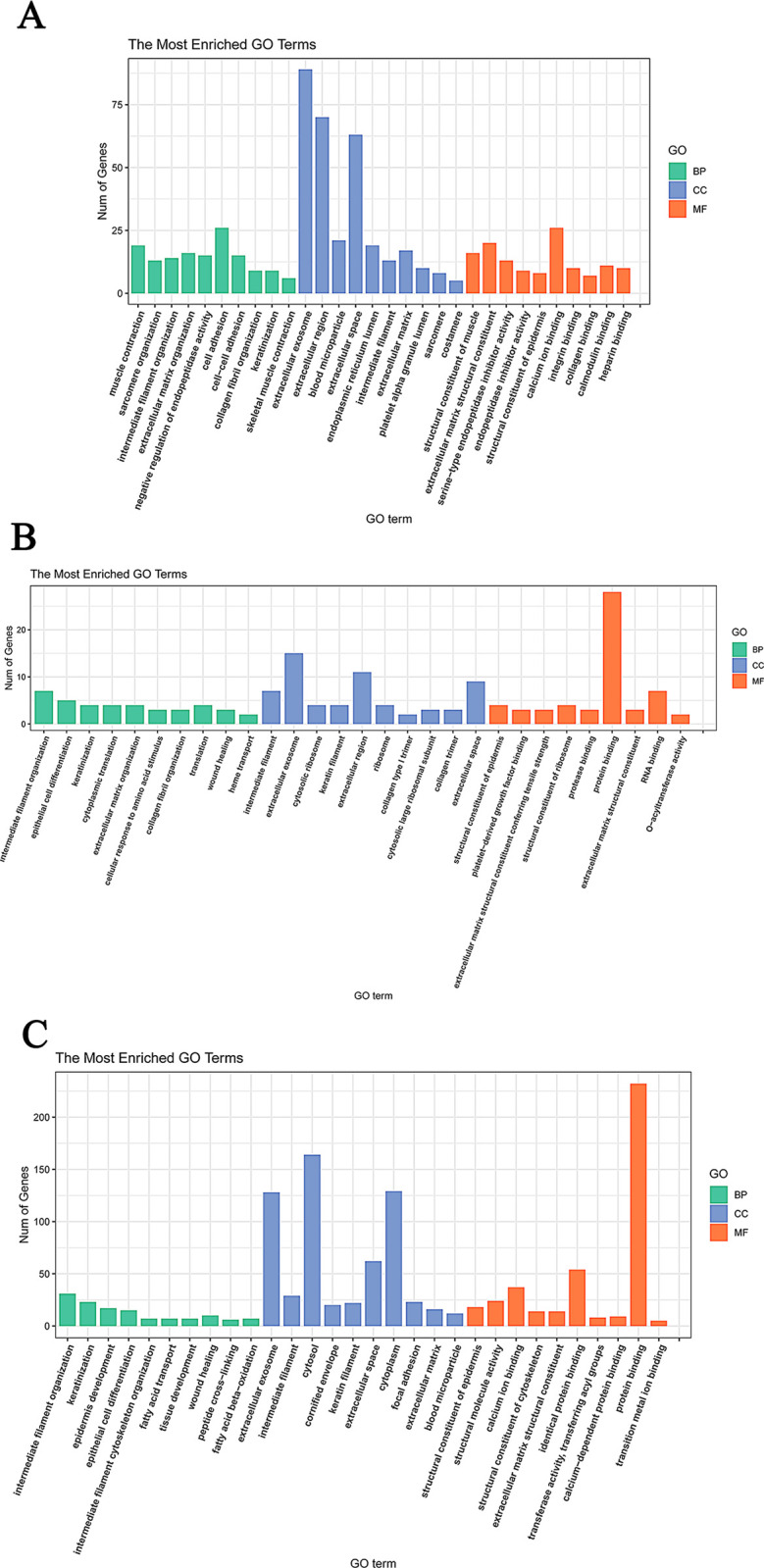
Top 10 DEPs according to GO function. (A) E75 vs E85; (B) E85 vs E95; (C) E95 vs E105.

The GO functional enrichment and the biological characteristics of HF development identified 13 terms potentially associated with SF development and 23 terms potentially associated with re-differentiated SF development. The DEPs identified in E75 vs E85 were most enriched in the extracellular exosome in the CC category, protein binding in the MF category, and cell adhesion in the BP category (Fig 4). The DEPs in the E85 vs E95 comparison were most enriched in the extracellular exosome (the CC category), protein binding (the MF category), and intermediate filament organization (the BP category). Lastly, the DEPs in the E95 vs E105 comparison were most enriched in the cytosol (the CC category), protein binding (the MF category), and intermediate filament organization (the BP category). These findings indicate that the corresponding functional categories were closely related to SF and skin development.

**Fig 5:**
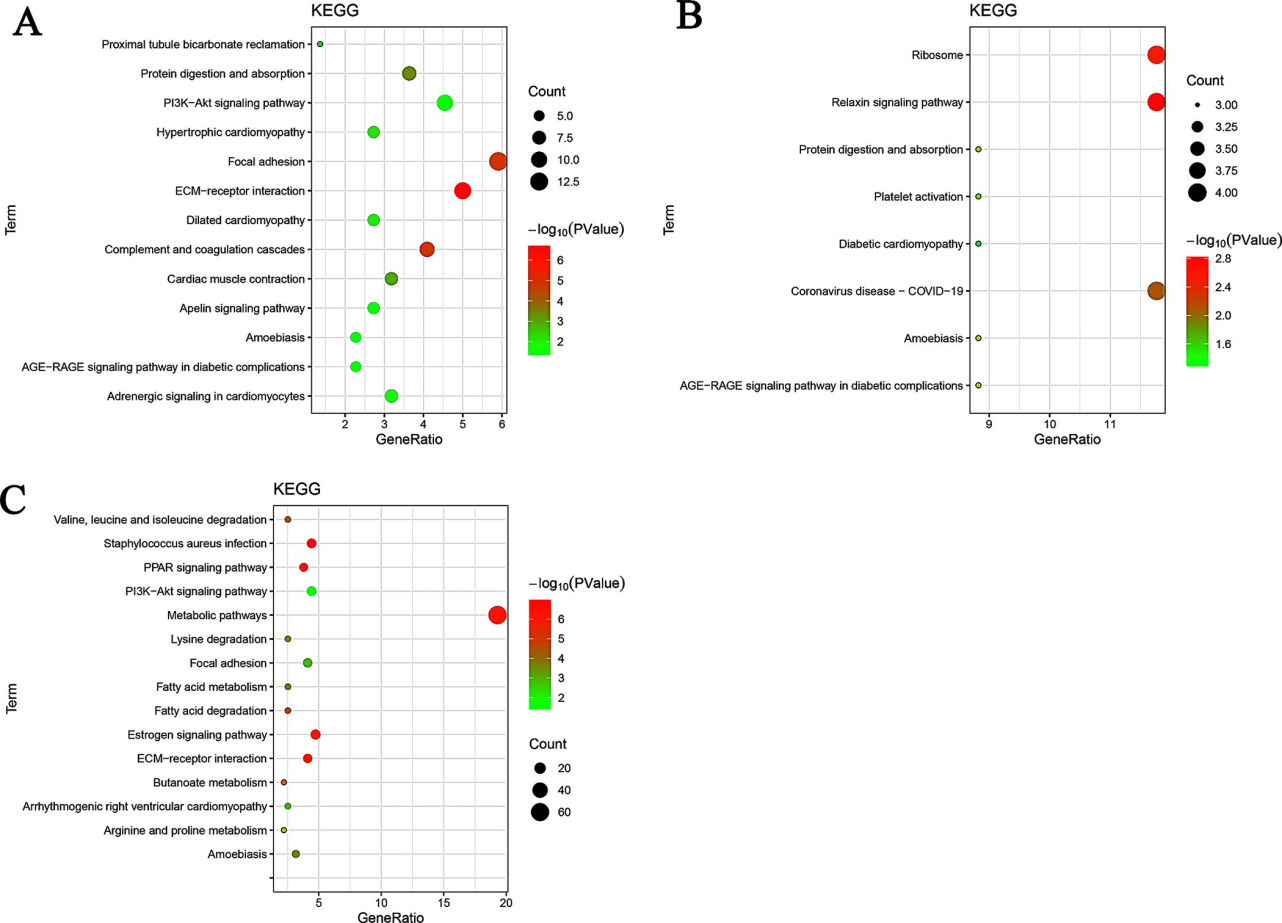
Scatter plot of KEGG pathway enrichment of DEPs. (A) E75 vs E85; (B) E85 vs E95; (C) E95 vs E105.

Furthermore, the DEPs in the comparison of E75 vs E85 were primarily involved in the PI3K−Akt signaling pathway, complement and coagulation cascades, and ECM−receptor interactions (Fig 5). In E85 vs E95, the main KEGG pathways involved the ribosome, protein digestion and absorption, and the relaxin signaling pathway. Lastly, in E95 vs E105, the major pathways included the PI3K−Akt signaling pathway, the estrogen signaling pathway, and ECM−receptor interactions. Notably, the DEPs in the comparisons of E75 vs E85 and E95 vs E105 were significantly enriched in the PI3K−Akt signaling pathway and ECM−receptor interactions. These pathways could therefore play crucial roles in the development of SFs. We conducted subsequent KEGG pathway enrichment analyses for the DEPs potentially associated with SF and re-differentiated SF development. The analyses revealed that 11 pathways were enriched in SF development, while 14 pathways were enriched in re-differentiated SF development (Table 3). There were 23 and 19 DEPs identified during the SF and re-differentiated SF development periods, respectively.

**Table 3:** DEPs related to SF and re-differentiated SF development involved in the KEGG pathways.

Period	Pathway ID	Pathways	Proteins	Counts	*P*-Value
E75 vs E85	oas04512	ECM-receptor interaction	COL1A1, VTN, FRAS1, TNXB, SDC4, SPP1, TNC, LAMC2, THBS4, THBS3	10	1.34E-11
	oas04510	Focal adhesion	COL1A1, VTN, TNXB, SPP1, TNC, LAMC2, THBS4, THBS3	8	5.09E-06
	oas04974	Protein digestion and absorption	DPP4, COL1A1, COL3A1, COL14A1, COL7A1, COL12A1, ATP1B1	7	5.15E-06
	oas04151	PI3K-Akt signaling pathway	COL1A1, VTN, TNXB, SPP1, TNC, LAMC2, THBS4, THBS3	8	2.86E-04
	oas04514	Cell adhesion molecules	CDH5, VCAN, SDC4, JAM3	4	1.81E-02
E95 vs E105	oas04512	ECM-receptor interaction	COL1A1, LAMA5, COL1A2, LAMA2, COL6A2, COL6A1, LAMA3, SDC1, COL6A3, LAMC2, ITGA6, THBS4	12	1.55E-12
	oas04510	Focal adhesion	COL1A1, LAMA5, COL1A2, LAMA2, ACTN1, COL6A2, COL6A1, LAMA3, COL6A3, LAMC2, ITGA6, THBS4	12	1.10E-08
	oas04915	Estrogen signaling pathway	KRT19, KRT18, KRT28, KRT27, KRT26, KRT25, KRT13, KRT35, KRT32, KRT10	10	6.62E-08
	oas04151	PI3K-Akt signaling pathway	COL1A1, LAMA5, COL1A2, LAMA2, COL6A2, COL6A1, LAMA3, COL6A3, LAMC2, ITGA6, THBS4	11	3.98E-05
	oas04974	Protein digestion and absorption	COL1A1, COL1A2, COL6A2, COL6A1, COL6A3	5	5.97E-03

### qPCR analysis of DEPs

**Fig 6:**
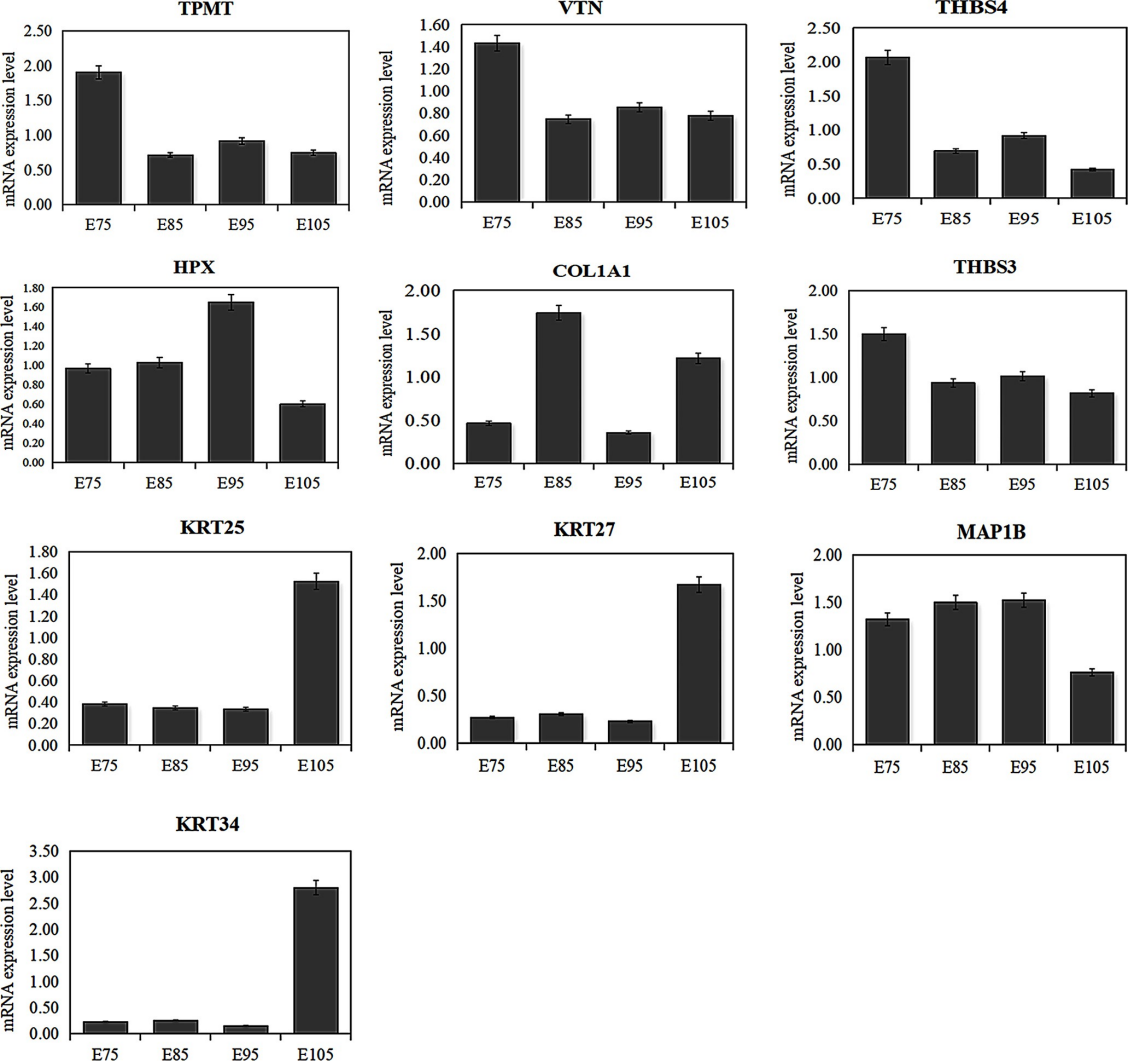
Expression and analysis of DEPs associated with SF and re-differentiated SF development.

We selected 10 DEPs to validate the results of proteomic analyses. The qPCR results revealed that the mRNA expression of the 10 genes was consistent with protein expression patterns, and this further supported the accuracy of the TMT-based proteomics data obtained in the HF development experiments (Fig 6).

### Western blot

**Fig 7:**
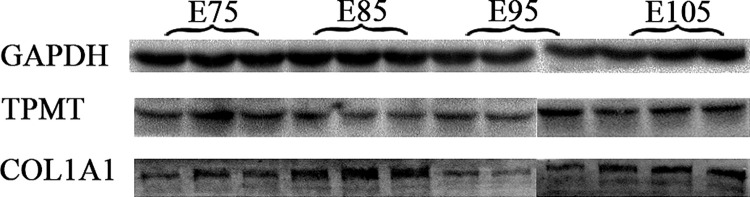
Protein expression confirmation by western blot.

Western blot analyses were performed to verify the expression determined for the 2 proteins under consideration (TPMT, COL1A1). The protein expression changes observed in the immunoblots were consistent with the TMT-based proteomics (Fig 7).

### PPI network of DEPs

**Fig 8:**
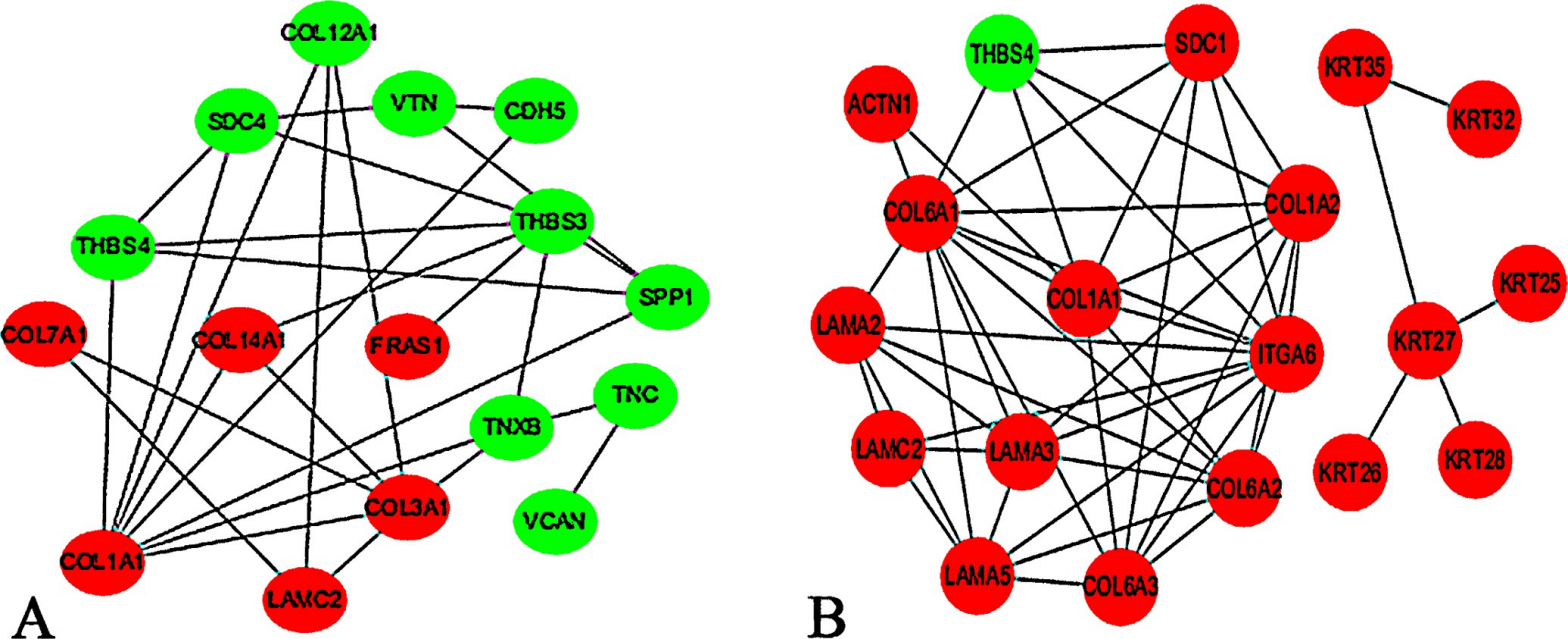
PPI network analysis of DEPs. (A) The PPI network of DEPs associated with SFs. (B) The PPI network of DEPs associated with re-differentiated SFs. A schematic representing the interaction network constructed for protein-coding DEPs. Green nodes indicate down-regulated proteins, and red nodes indicate up-regulated proteins. The lines indicate the interactions between the proteins.

We conducted PPI network analyses for the DEPs associated with the GO and KEGG pathways. Notably, six up-regulated proteins (COL1A1, et al.) and 10 down-regulated proteins (VCAN, et al.) existed in one interaction network related to SF development in the comparison of E75 vs E85 (Fig 8A), and 18 up-regulated proteins (COL6A1, et al.) and a single down-regulated protein (THBS4) existed in one interaction network in E95 vs E105 (Fig 8B). Importantly, COL1A1 and LAMC2 were up-regulated, whereas THBS4 was down-regulated in both the E85 vs E75 and E105 vs E95 comparisons, suggesting that the three genes played similar roles in the development of SFs and re-differentiated SFs.

## Discussion

Wool is both a product and a fundamental raw material in the textile industry. Wool fibers develop from the differentiation of the ectoderm of the skin, and this process involves the formation of sebaceous glands, sweat glands, nails, and other accessory structures [17]. HF development begins during fetal development, and the S/P ratio is an important index reflecting the state of FD [18]. Selecting individuals with a higher S/P value can not only increase wool yield but also improve the wool length and fineness [19].

The morphology of HFs has been extensively studied; Xu et al. confirmed through tissue sections that primitive HFs begin to appear at 95 gestational days (E95) in Hu sheep [20]. In Subo Merino sheep, SF initiation occurs between E80 and E85, while re-differentiated SF production begins between E105 and E135 [21]. In Chinese Merino sheep (Junken type), SF initiation occurs at E85, and re-differentiated SFs appear at E105 [1]. In Chinese Superfine Merino sheep (Gansu type), the SF density gradually increases from E87 to E117, reaching a maximum of 232.8 ± 12.44/mm^2^ at E117 and then gradually tapering off; the S/P ratio increased gradually from E87 to E126, peaking at 9.96 on E126 [22]. In this study, we confirmed that SF production appeared on E85, while re-differentiated SFs were first observed at E105, consistent with previous reports [6].

Previous studies have investigated miR-1-3p [23], miR-767 [24], WNT5a [25], VEGF [26], KRT5 [27], β-catenin [28], the Wnt signaling pathway [7], and the BMP pathway [29] for their association with HF development. In this study, TMT based comparative proteomic strategy was used to quantitatively identify the DEPs between different SF developmental periods, and a total of 509 proteins were found to be differentially expressed. In order to verify the DEPs identified by TMT LC-MS/MS analysis, qPCR and western blot were performed to determine the gene expression change at transcription and translation level. Our results showed that the expression levels of 10 genes were consistent with those of the TMT LC-MS/MS analysis, to ascertain the reliability of the TMT data. The TPMT and COL1A1 mRNA expression level was consistent with the protein level from each developmental period. Furthermore, we performed KEGG pathway analysis on DEPs associated with the morphogenesis of SFs and re-differentiated SFs. The ECM-receptor interaction, focal adhesion, protein digestion and absorption, and PI3K-Akt signaling pathway had the highest protein enrichment levels during the development of SFs and re-differentiated SFs. Interestingly, cell adhesion molecules were enriched exclusively during the initiation of SFs, while the estrogen signaling pathway was enriched solely during the initiation of re-differentiated SFs. We identified 509 DEPs, 39 of which were actively associated with wool growth and the development of SFs and re-differentiated SFs. These included COL1A1, THBS3, ITGA6, COL6A1, and THBS4, all of which enriched in the ECM-receptor interaction pathway. The ECM-receptor pathway is involved in regulating HF development in cashmere goats [30, 31]. COL1A1 encodes the pre-alpha 1 chain of type I collagen, a protein abundant in various tissues, including bone, skin, and tendons that plays a crucial role in the development of most animals [32]. Additionally, COL1A1 is involved in numerous physiological processes, including ECM synthesis and cell proliferation and migration; as such, it promotes the rapid growth of epithelial cells [33, 34]. Gene co-expression analysis and proteome sequencing have identified COL1A1 as a key factor associated with the growth of SFs [35, 36]. Specifically, COL1A1 can be induced by cell adhesion and transport dynamics to enhance cell aggregation for the formation of dermal papillae [30] and thereby promote the growth of SFs. Additionally, single-cell transcriptome sequencing analysis has implicated COL1A1 in the fineness of cashmere goat villi [37]. However, few studies have illustrated the relationship between COL1A1 and HF development. In our study, the expression of COL1A1 was significantly increased at E85 and E105. Our findings suggest that the ECM-receptor interaction may play a crucial role in regulating the initiation of SFs through the involvement of COL1A1.

PI3K-Akt is an important factor in diverse cellular activities. In mammalian cells, activation of Akt induces cell proliferation and survival, while over-activated Akt signaling tends to induce cell transformation [38–40]. One study identified the crucial involvement of the PI3K-Akt signaling pathway in *de novo* HF regeneration [41]. Melatonin can enhance the proliferation of dermal papilla cells and improve cell viability via activation of the PI3K-Akt signaling pathway, thereby participating in the regulation of the HF life cycle [42]. In our study, COL1A1, THBS3, ITGA6, COL6A1, and THBS4 were significantly enriched in the PI3K-Akt signaling pathway, suggesting a strong interrelationship. THBS4 [43, 44] and ITGA6 [45, 46] are involved in the function of the PI3K-Akt signaling pathway. THBS3 and THBS4 are members of the THBS family, a group of glycoproteins that are secreted by activated platelets [47] and expressed on the skin of sheep [48]. Several studies have elucidated the significant physiological roles played by the THBS family in the processes of angiogenesis, cancer progression, inflammation, immune modulation, fibrosis regulation, and the maintenance of myocardial integrity and function [49–52]. However, to date, there have been no investigations into the involvement of THBS3 or THBS3 in regulating SF development in sheep. In our study, the expression of THBS4 was significantly decreased at E85 and E105, while the expression of THBS3 was significantly decreased only at E85. We hypothesize that modulation of the PI3K-Akt signaling pathway may regulate the development of SFs and re-differentiated SFs by down-regulating the expression of THBS4 and THBS3. ITGA6 is a transmembrane protein involved in cell surface adhesion and signaling as well as in the regulation of proliferation, tumor invasion, and metastasis [53]; similar to THBS3 and THBS4, ITGA6 is also expressed in the skin of sheep [48]. Moreover, ITGA6 serves as one of the key marker genes for HF stem cells within the skin tissue [54]. Notably, several studies have identified miR-143 as a potential regulator of ITGA6 expression during the transition from the SF degenerative phase to the resting phase by inhibiting its translation [55]. It has been postulated that this signaling cascade governs the development of re-differentiated SFs by up-regulating ITGA6; however, further investigation should be conducted to determine the regulatory mechanisms of SF development.

Estrogen exerts its effects primarily through interactions with the estrogen receptors (ERs). Estrogen modulates HF growth and cyclical regulation by binding to locally expressed high-affinity ERs [56]. In this study, we observed a significant enrichment of the estrogen signaling pathway, with most of the enriched genes being keratins, proteins that may be involved in regulating HF development. In summary, these findings indicate that many skin-specific DEPs influence SF development. Further studies are needed to characterize the functional roles of proteins related to SF development in fine-wool sheep.

In summary, we used proteomics data to construct a protein expression profile throughout SF development. We screened a series of candidate proteins and signaling pathways that may participate in SF development via bioinformatic analyses. These data and results advance the limited body of proteomics research in Chinese Merino sheep and significantly increase our understanding of the critical biological processes that occur during wool development. However, the mechanisms of action of signal transduction, apoptosis, nutrition, and regulatory factors in SF development require further study. Our plans in this area of research include the following: 1) verifying the predicted molecular mechanisms guiding SF growth and further investigating the interactions and functional relationships of the DEPs in SF development; 2) conducting a combined analysis of proteins and genes, incorporating the gene level, to comprehensively study the regulatory mechanisms underlying SF growth, and 3) culturing skin hair follicle cells to the growth phase and utilizing gene editing technologies to validate the specific roles of target proteins in SF growth. These future endeavors will provide new targets for breeding and improving the traits of super fine-wool sheep.

## Conclusions

TMT-based quantitative proteomics and LC‒MS/MS analysis identified 509 DEPs as being involved in SF and re-differentiated SF development, and 39 proteins, including COL1A1, THBS3, THBS4, ITGA6, and COL1A6, had potential roles in the initiation of SF and re-differentiated SF development. The proteins associated with each developmental stage were closely related to ECM–receptor interactions, focal adhesion, protein digestion and absorption, the PI3K-Akt signaling pathway, cell adhesion molecules pathways, and estrogen signaling pathways, indicating that these pathways may play important roles in initiation of SF and re-differentiated SF development. Our findings provide new insights into the molecular mechanisms involved in SF morphogenesis and suggest potential targets for breeding and improving wool traits in fine-wool sheep.

## Supporting information

S1 Table Significant DEPs among three comparative periods of sheep HF development. (XLSX)

S1 File Accessible data. (DOCX)

S1 Raw images (PDF)
