# Supplementary material for: TMT-based quantitative proteomics reveals the genetic mechanisms of secondary hair follicle development in fine-wool sheep
Source: PLoS One. 2025 Feb 6;20(2):e0315637. doi: 10.1371/journal.pone.0315637 (PMC11801579; doi:10.1371/journal.pone.0315637)
Supplement: S1 File — (DOCX) [file pone.0315637.s002.docx]

We have deposited the raw proteomics data in the PRIDE database. The mass spectrometry proteomics data have been deposited in the ProteomeXchange Consortium via the PRIDE partner repository with the dataset identifier PXD052470.

Unique link: <https://www.ebi.ac.uk/pride/review> dataset/99e10a0b5dde44aab6e2f0920f58f6f7

Project accession: PXD052470

Token: nJtRCScXzNAQ
