# Supplementary material for: TMT-based quantitative proteomics reveals the genetic mechanisms of secondary hair follicle development in fine-wool sheep
Source: PLoS One. 2025 Feb 6;20(2):e0315637. doi: 10.1371/journal.pone.0315637 (PMC11801579; doi:10.1371/journal.pone.0315637)
Supplement: S1 Raw images — (PDF) [file pone.0315637.s003.pdf]

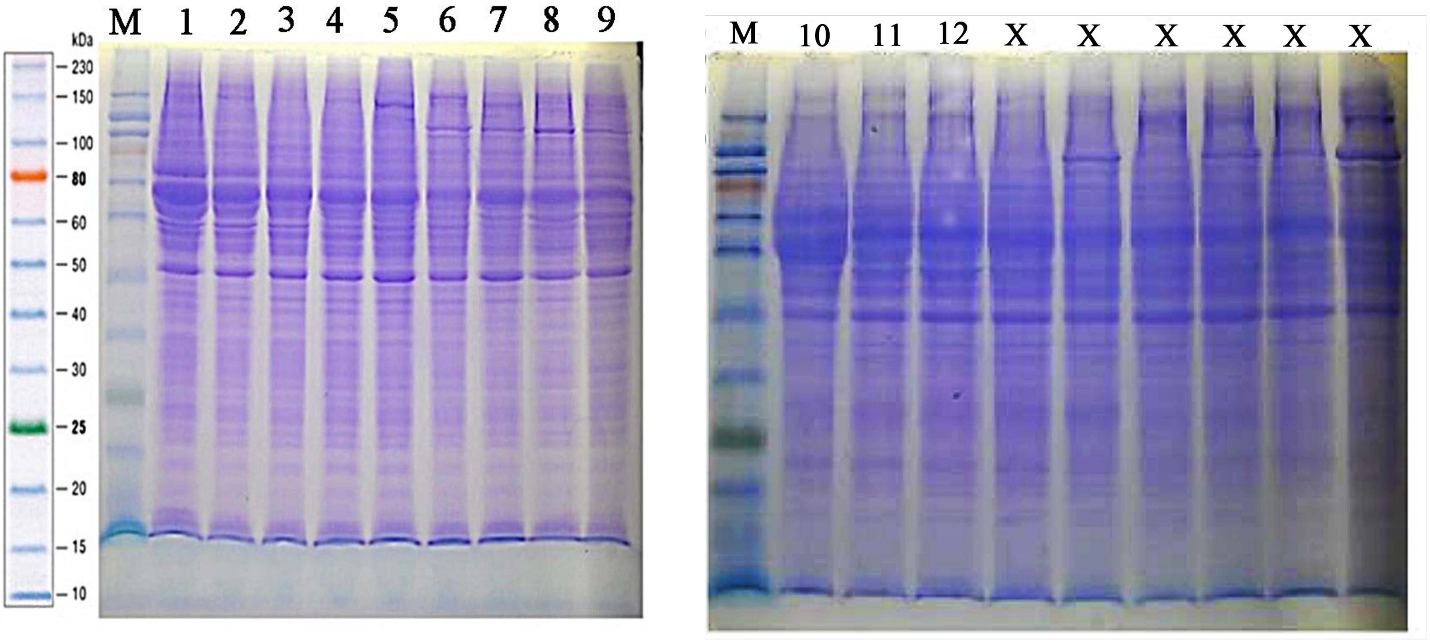

Figure 2. SDS-PAGE gel electrophoresis raw blot.
M: the protein molecular weight standard. 1-3: E75. 4-6: E85.
7-9: E95. 10-12: E105. X: lanes not included in the final figure

Figure 7. western blot raw blot.

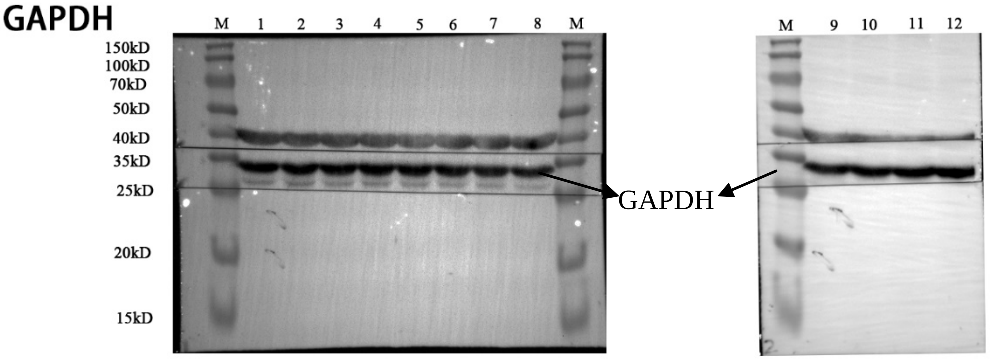

GAPDH WB complete PVDF picture（Maker:15-150kD; Lane1-3:E75; Lane 4-6:E85; Lane7-9:E95; Lane 10-12:E105

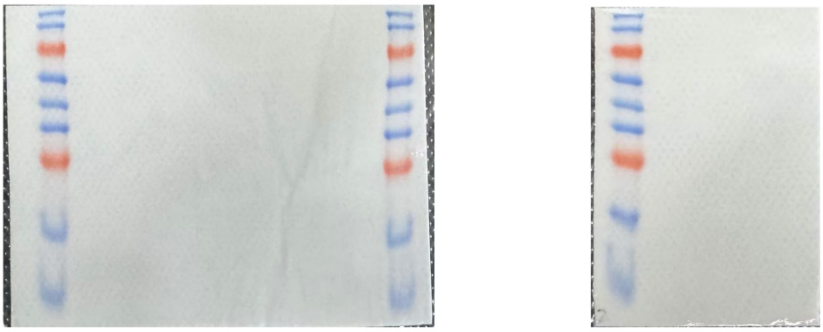

GAPDH WB complete PVDF membrane

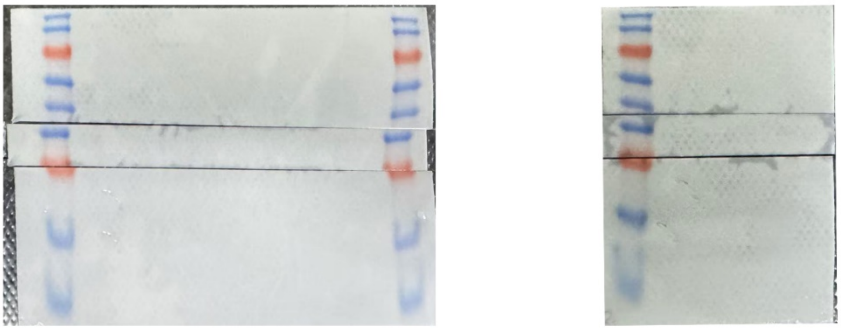

Cut the PVDF membrane and incubate it with GAPDH primary antibody

TPMT

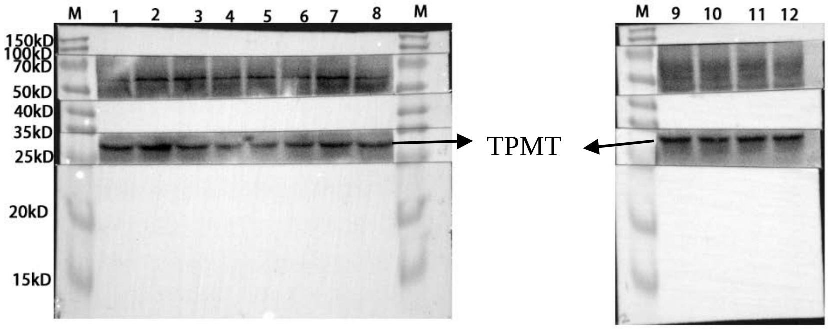

TPMT WB complete PVDF picture（Maker:15-150kD; Lane1-3:E75; Lane 4-6:E85; Lane7-9:E95; Lane 10-12:E105

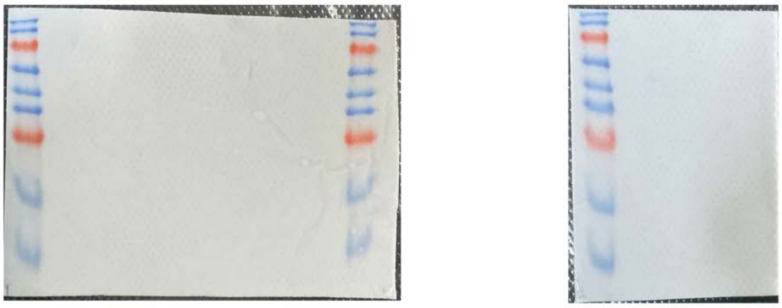

TPMT WB complete PVDF membrane

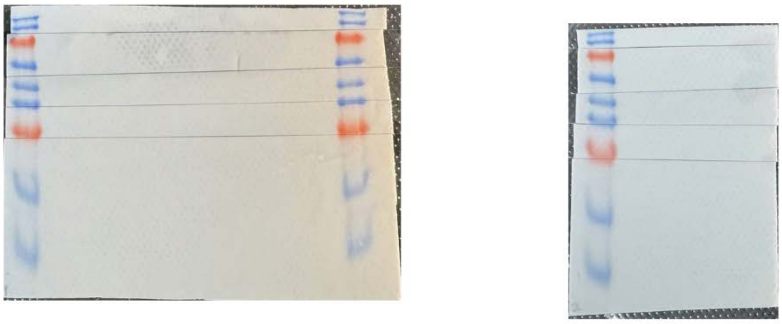

Cut the PVDF membrane and incubate it with TPMT primary antibody

COL1A1

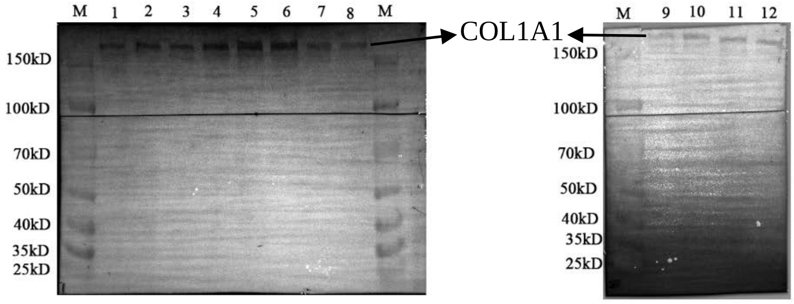

COL1A1 WB **complete PVDF picture（Maker:15-150kD; Lane1-3:E75; Lane 4-6:E85; Lane7-9:E95; Lane 10-12:E105**

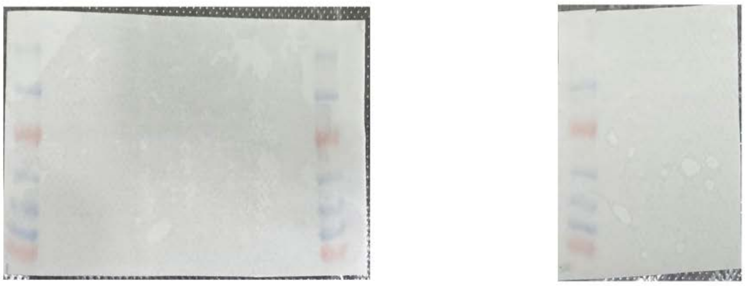

COL1A1 WB complete PVDF membrane

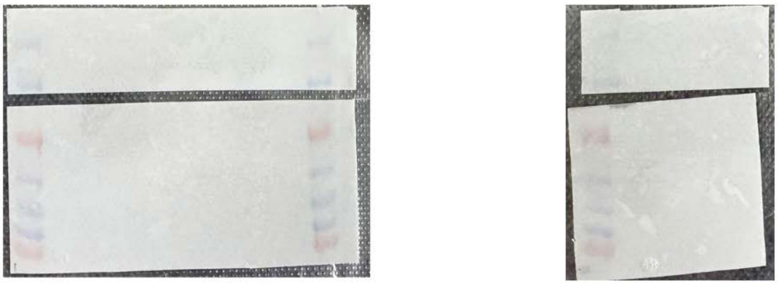

Cut the PVDF membrane and incubate it with COL1A1 primary antibody
